# Exogenous Cortisol Administration; Effects on Risk Taking Behavior, Exercise Performance, and Physiological and Neurophysiological Responses

**DOI:** 10.3389/fphys.2016.00640

**Published:** 2016-12-27

**Authors:** Caroline V. Robertson, Maarten A. Immink, Frank E. Marino

**Affiliations:** ^1^School of Exercise Science, Sport and Health, Faculty of Science, Charles Sturt UniversityBathurst, NSW, Australia; ^2^School of Health Sciences, Alliance for Research in Exercise, Nutrition and Activity (ARENA) and Cognitive Neuroscience Laboratory, University of South AustraliaAdelaide, SA, Australia

**Keywords:** risk taking, exogenous cortisol, neurophysiological responses, BART test, exercise performance

## Abstract

**Rationale:** Exogenous cortisol is a modulator of behavior related to increased motivated decision making (Putman et al., 2010), where risky choices yield potentially big reward. Making risk based judgments has been shown to be important to athletes in optimizing pacing during endurance events (Renfree et al., 2014; Micklewright et al., 2015).

**Objectives:** Therefore, the aims of this study were to examine the effect of 50 mg exogenous cortisol on neurophysiological responses and risk taking behavior in nine healthy men. Further to this, to examine the effect of exogenous cortisol on exercise performance.

**Methods:** Using a double blind counterbalanced design, cyclists completed a placebo (PLA), and a cortisol (COR) trial (50 mg cortisol), with drug ingestion at 0 min. Each trial consisted of a rest period from 0 to 60 min, followed by a risk taking behavior task, a 30 min time trial (TT) with 5 × 30 s sprints at the following time intervals; 5, 11, 17, 23, and 29 min. Salivary cortisol (SaCOR), Electroencephalography (EEG) and Near Infrared Spectroscopy (NIRs) were measured at 15, 30, 45, and 60 min post-ingestion. Glucose and lactate samples were taken at 0 and 60 min post-ingestion. During exercise, power output (PO), heart rate (HR), EEG, and NIRS were measured. SaCOR was measured 10 min post-exercise.

**Results:** Cortisol increased risk taking behavior from baseline testing. This was in line with significant neurophysiological changes at rest and during exercise. At rest, SaCOR levels were higher (*P* < 0.01) in COR compared to PLA (29.7 ± 22.7 and 3.27 ± 0.7 nmol/l, respectively). At 60 min alpha slow EEG response was higher in COR than PLA in the PFC (5.5 ± 6.4 vs. −0.02 ± 8.7% change; *P* < 0.01). During the TT there was no difference in total km, average power or average sprint power, although Peak power (PP) achieved was lower in COR than PLA (465.3 ± 83.4 and 499.8 ± 104.3; *P* < 0.05) and cerebral oxygenation was lower in COR (*P* < 0.05).

**Conclusion:** This is the first study to examine the effect of exogenous cortisol on exercise performance. These results are in line with previous research showing altered risk taking behavior following exogenous cortisol, however the altered behavior did not translate into changes in exercise performance.

## Introduction

Cortisol is generally associated with mediating the primary effects of stress such as aversive and avoidance inducing behavior (Marinelli and Piazza, 2002) and is widely used as a biomarker for stress (Hellhammer et al., 2009). Conversely, the provision of exogenous cortisol has recently been shown to be a modulator in processing of threat related stimuli, such as reduced fearful symptomology in those with post-traumatic stress disorder (PTSD; Putman et al., 2007). Exogenous cortisol has also been shown to increase motivated decision making behavior in humans (Putman et al., 2010). Further to this, animal models have shown that high levels of glucocorticoids (GCs) increase reward and approach related behavior (Piazza et al., 1996a; Marinelli et al., 1998; Mikics et al., 2007), produce greater locomotor activity and higher levels of impulsivity (Koob and Bloom, 1988; Piazza et al., 1991, 1996b). The mechanisms by which exogenous cortisol may alter reward and approach related behavior has been postulated to be due to increased dopamine (DA) activity (Marinelli and Piazza, 2002) which occurs when stress levels of GCs induce an increased release of DA from the nucleus accumbens (Rougé-Pont et al., 1998), a component of the brain's reward system (Roitman et al., 2005).

Within an athletic situation, making risk based judgments has been proposed to be important to athletes in optimizing pacing during endurance events (Micklewright et al., 2015). Not only is it important to ensure that pacing is optimized but, in achieving the fastest overall speed possible, pace at the end of the event is not compromised resulting in a slower overall time. In addition to this, during a race or event, competitors are subject to further risk evaluation situations (Renfree et al., 2014; Micklewright et al., 2015). Exposure to external environmental conditions may alter the decisions and calculations that are being made by the brain in relation to pacing. The presence of opponents and the necessity to respond to them, creates the need to weigh up all possible behavioral choices, relating to a change in pace, and their associated benefits and risks (Renfree et al., 2012, 2014).

An individual's propensity to take risks and its effect on performance, pacing and RPE, has recently been examined (Micklewright et al., 2015). Cyclists with a higher perception of risk showed a slower starting pace and perception of risk was also shown to be associated with deviations from a predicted pace (Micklewright et al., 2015). Ultra-marathon runners were also investigated revealing the same relationship with greater risk perception, associated with slower starting pace (Micklewright et al., 2015). Whilst risk played a role in pacing strategy, their overall race time in both events examined was the same. Calculation of a hazard score has also shown that a fast start elevates the perception of hazard compared to a slow start (Williams et al., 2015) but again with no change in TT performance. These data show that a greater perception of risk results in slower starting strategies which is likely due to the risk assessment of having to get to the end of the race with less chance of a catastrophic collapse (de Koning et al., 2011). As of yet the internal manipulation of risk behavior via exogenous cortisol and the impact on exercise performance has yet to be examined.

Therefore, the aims of this study were to examine the effect of exogenous cortisol on neurophysiological responses and risk taking behavior. Further to this, to examine the effect of exogenous cortisol on time trial performance. The hypothesis was that risk taking behavior would be enhanced with exogenous cortisol and that this would also influence participants approach to exercise, with a less risk adverse approach.

## Experimental procedures

### Subjects and ethical approval

Eleven, healthy, trained, male cyclists from local cycling and triathlon clubs were recruited for this study. Participants were required to have been training for at least 2 years and to be currently training for a minimum of 2 h a week. Each participant was paid a small amount on completion of the study, as an incentive to participate. Their mean ± *SD* age, height, weight, $\dot{V}O_{2}\;$ max and maximal aerobic peak power were, respectively; 39.6 ± 10.4 years, 183.7 ± 8.2 cm, 83.6 ± 14.0 kg, 4.4 ± 0.6 l min^−1^, 397.1 ± 36.3 W. After being informed of the risks associated with the experiment each participant signed a letter of informed consent. Subjects were cleared by a physician for any contraindications of consuming 50 mg of cortisol as defined by the medical practitioner; which included neurological diseases as well as dysfunction of the adrenal glands. The study was approved by the Charles Sturt University Research and Ethics Committee and conformed to standards set by the latest revision of the Declaration of Helsinki.

### Procedure

Participants attended the laboratory on three different occasions; baseline and two intervention trial visits. At each intervention trial, participants were given either 50 mg of cortisol (COR) or a placebo (PLA). The experimental design was double blinded, randomized and counterbalanced. All test sessions were scheduled to take place between 1300 and 2000 h to reduce diurnal variations of the cortisol response (Putman et al., 2010) and at a time when hormone levels are relatively stable (Henckens et al., 2012). Each participant was tested at the same time of day for all visits. Prior to each visit subjects were asked to refrain from any exercise for at least 24 h prior to testing and to undertake only easy training from 36 h prior to each visit. All participants maintained a regular diet during the study period and were asked to refrain from alcohol ingestion for at least 24 h prior to testing and caffeine 6 h prior. Participants were required to not consume any food or drink with the exception of water from 1.5 h prior to arriving at the laboratory. Prior to each visit subjects were provided with a food diary to complete which ensured that meals were consumed at the same time of day to avoid interaction with diurnal changes in cortisol and food intake (Follenius et al., 1982). This also served to ensure that macronutrient intake was kept the same to maintain high levels of muscle glycogen for exercise performance.

## Baseline and initial screening

As part of an initial screening process, participants collected salivary cortisol measures on the morning of their baseline test to determine cortisol awakening response (CAR). Samples were collected on awakening, and at 30, 45, and 60 min post-wakening. Samples were collected by placing oral swabs under the front of the tongue for 2 min before being placed into salivette tubes (Salimetrics, State College, PA, USA) and refrigerated. Participants were instructed to only drink water during this period and for nothing to be consumed 10 min prior to each sample. These instructions were explained verbally as well as via written instructions provided with the salivettes and swabs. After collection samples were frozen at −20°C until analysis.

This was completed to ensure that participants did not have dysfunctional cortisol regulation. On the afternoon of the same day, participants came into the lab to complete a baseline (BASE) visit including an initial Balloon Analog Risk Task (BART) described subsequently (Lejuez et al., 2002), and a maximal oxygen uptake test to establish fitness levels.

### Balloon analog risk task (BART)

Reliability of the BART test has been shown previously both within (Lejuez et al., 2002) and across (Lejuez et al., 2003a) task administrations. The test has also been validated for risk taking behavior in adolescents (Lejuez et al., 2003b) and risk taking behaviors such as drug use (Lejuez et al., 2004) and delinquent activity (Aklin et al., 2005). Participants completed the computer based test and were blinded to the rationale behind the test so that they could not manipulate the results.

The computer screen showed a small simulated balloon accompanied by a balloon pump, a button labeled *Collect $$$*, as well as a “money earned” display which informed the participants of the running total of money earned to that point. Participants were blinded from the amount earned per balloon. This was to ensure that they could not judge how many times they had pumped up the balloon. Each participant was faced with 30 balloons which were set to explode randomly. When each balloon was pumped past its set point, it exploded and no money was accrued and a new balloon was presented. If the *Collect $$$* button was pressed before the balloon exploded, the participants earned the money from the number of pumps on completed on that balloon. At any point during each balloon trial, the participant could stop pumping the balloon and click the “collect $$$” button.

### Exercise tests

#### Maximal oxygen uptake

Participants were fitted with a facemask (Hans Rudolph Kansas City, MO) connected to a rapid response gas analyser (AEI Technologies, Pittsburgh, PA, USA) for the measurement of pulmonary gas exchange. A 1 min resting sample was collected before the initiation of the test. Cyclists performed a cycle ramp test to exhaustion on a Lode cycle ergometer consisting of a ramp increase in power output of 30 W min^−1^ (1 W every 2 s) until volitional exhaustion. Participants were asked to maintain their preferred cadence (all participants selected between 80 and 100 rpm) for as long as possible. The test was terminated voluntarily by each subject when they could no longer sustain the required power output whilst staying seated in the saddle. The test was performed in a quiet room with no internal or external interruptions and no verbal encouragement was provided during the test.

Pulmonary gas exchange was measured breath-by-breath throughout the exercise test using a custom-designed expired gas analysis metabolic system. All data were acquired using custom-developed software (LabVIEW; National Instruments, Austin, TX, USA) and commercial electronic acquisition devices (National Instruments).

#### Thirty minute cycle test familiarization

Following a rest period of at least 15 min participants were then required to complete 15 min of the 30 min protocol to ensure that they were familiar with the bike gearing and the requirement for sprinting during an all-out time trial type effort.

## Intervention trials

Each participant also completed two intervention visits, in addition to the BASE visit, as described subsequently. All test days were completed within 15 days with at least 2 days between trials.

### Resting measurements

**Figure 1:**
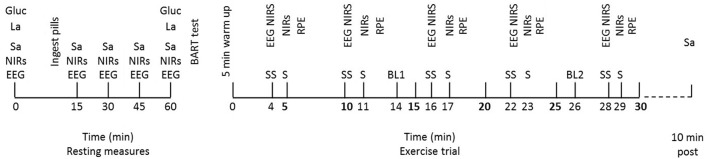
**Schematic representation of the intervention trials with resting and exercise measures at their relevant time points**. Physiological measures at rest; EEG and NIRS, salivary cortisol (Sa), blood glucose (gluc), and blood lactate (La). Balloon Analog Risk Task (BART) completed following all resting measures. Measures during exercise at steady state (SS) time points; NIRs, EEG. Measures during each sprint (S); NIRs. Blood samples (glucose and lactate) taken at two time points throughout, BL1 and BL2. Salivary cortisol (Sa) taken at 10 min post-exercise.

On arrival at the laboratory subjects were seated and made comfortable. They were instructed that they were allowed to drink water during the resting period as long as they did not drink within 10 min of a saliva sample. Following this they were fitted with a wireless EEG headset (B-Alert, ABM, Ca) and two near-infrared spectrometer (NIRs) probes (NIRO-200, Hamamatsu, Japan), outlined subsequently. EEG and NIRs measures were taken at rest. Subjects also provided a resting saliva sample, as described above. Capillary blood samples were provided for the determination of blood lactate and glucose as described subsequently. Following initial resting measures (at 0 min), subjects were given two capsules to take, containing either 50 mg cortisol (COR; Cortate, Aspen Pharmacare, NSW, Aus) or placebo (PLA). This dose was chosen as it is the equivalent to the highest dose given to humans in a research environment (van Peer et al., 2008). The EEG, NIRS, and salivary resting measures were repeated at the following time points post-capsule ingestion; 15, 30, 45, and 60 min. A second blood sample was also taken at 60 min post-ingestion. Following this, the participants were asked to complete a BART test, as described above (see Figure 1).

### Exercise trials

Following completion of the BART test, subjects were transferred to the cycle ergometer (Racermate, Velotron, WA, USA) and provided with a standardized 5 min 100 W warm up before beginning the trial. Bike set up was replicated as per their BASE trial. Each exercise trial consisted of a 30 min effort with 30 s sprints at the following time intervals; 5, 11, 17, 23, and 29 min. Sprints were included in the protocol to allow potential access to higher levels of work regulation which might appear with the cortisol ingestion. During the 30 min trial, participants were blinded to all feedback with the exception of time. This ensured that they were unaware of their performance level between trials, but that the exercise bout was closed loop allowing self-pacing. Each participant was asked to approach the exercise bout as they would a race and to complete as much distance as possible in the 30 min.

During exercise, power output (W), distance (km), and heart rate were recorded continuously. EEG and NIRS samples were collected for 30 s, 1 min prior to each sprint at the following steady state (ss) time points; 4, 10, 16, 22, and 28 min. During this collection period, participants were asked to keep their eyes closed to reduce eye blink noise and to maintain as stable a cycling posture as possible in order to avoid excess movement artifact. During each 30 s sprint NIRS data were collected. No EEG data were collected at this time due to the noise associated with collecting EEG during high intensity exercise. Following each sprint an RPE score was obtained. Exercise blood samples were collected via finger prick capillary technique at 14 min (BL 1) and 26 min (BL 2); which equated to approximately half way through the protocol and as near to the end as possible without interfering with the other measurements taking place. Immediately post-exercise, subjects were permitted to have one drink of water if they wished and 10 min post-exercise a further salivary cortisol sample was taken (see Figure 1).

## Measurements

### Heart rate

Heart rate in beats.min^−1^ (HR) was measured and recorded with a chest strap and monitor (Polar RS400, Kempele, Finland) continuously throughout the trials.

### Rating of perceived exertion (RPE)

As an index of perceived exertion, the RPE was assessed using the Borg scale (Borg, 1990) where rankings ranged from 6 (no exertion at all) to 20 (maximal exertion). RPE ratings were taken at the end of each sprint and denoted as the following time points; RPE 1, 2, 3, 4, and 5.

### Blood collection and biochemical analysis

For each resting and exercise blood draw a 100 μL sample of capillary blood was collected from a hyperemic fingertip for analysis of blood lactate and glucose (ABL825 Radiometer, Copenhagen, Denmark).

### Neurophysiological measurements

#### Electroencephalography (EEG)

Subjects were fitted with a 20 channel, 256 Hz, wireless electroencephalography (EEG) headset (B-Alert, ABM, Ca). Measurements for EEG strap size were taken; Sagittal, coronal plane, and circumferential measures were taken for correct strip size and EEG placement using the 10–20 international system (Rowan and Tolunsky, 2006). Marker pen was used for placement of marks on the forehead at 10% of the coronal measure from the nasion to the inion for placement of FP1 and FP2, to ensure correct strip placement as defined by Jasper (cited in Rowan and Tolunsky, 2006). Synapse Conductive Electrode Cream (ABM, Ca) was used to increase conductance from scalp to electrode. The impedance of all electrodes was maintained below 40 kΩ as suggested by the manufacturer due to the hybrid active electrodes. Paired mastoid references were used and electrode and reference sites were cleaned and abraded prior to fitting. Data were sampled with a bandpass filter from 0.5 to 65 Hz (at 3 dB attenuation) obtained digitally with Sigma-Delta A/D converters. Data were acquired wirelessly across a RF link via an RS232 interface. The EEG signals were inspected continuously via online assessment at rest and during exercise. While EEG was recorded at all 20 electrode sites, data produced at the following sites were used for analysis; F3, F4, Fz, F7, F8, FP1, FP2, C3, C4, Cz.

#### Near-infrared spectroscopy (NIRS)

Two near-infrared spectrometer (NIRS) probes (NIRO-200, Hamamatsu, Japan) were placed over the left and right pre frontal lobes between FP1 and F3 (left) and FP2 and F4 (right; Billaut et al., 2010). The NIRS probes were adhered to the skin after the EEG strip had been put in place in accordance of the 10–20 international system to ensure accurate placement for each repeat trial. Each probe pair was placed in a black plastic probe holder, with predefined inter optode spacing of 4 cm. The larger inter optode distance was chosen to provide a greater maximum depth measured (Okada et al., 1995). The emission and detection probes were placed in opposite directions for each channel and the detection probes placed next to each other when on the forehead. The probes were housed in black rubber holders and attached to the skin with double sided adhesive discs. The probe holders were also secured in place by a black headband to reduce any extraneous light infiltration and to prevent any change in their position during exercise. Data were sampled at 1 Hz. Resting measurements of both EEG and NIRS were taken for 1 min with eyes closed (EC). This served to provide a baseline of 60 s to which other data points were normalized.

### Data analysis

#### Ventilatory parameters

Breath-by-breath gas exchange data from all $\dot{V}O_{2}\;$ max tests were transferred to a spreadsheet program (Microsoft Excel) for further analysis. All $\dot{V}O_{2}\;$ data were time averaged over eight breaths. $\dot{V}O_{2}max$ was determined by the highest serial average of eight breaths as has been performed previously (Robertson and Marino, 2015).

#### Power output and distance

Power output and distance were downloaded from the computer files generated by the cycle ergometer (Racermate, Velotron, WA, USA). Power output was averaged over the whole 30 m min trial, as well as the for each steady state period. Peak power and average power output were obtained for the sprints. Total distance completed was obtained from each file.

#### EEG analysis

EEG data were processed and analyzed using B-Alert lab (Version 2.0, ABM, Ca). Each 30 s sample was visually inspected for artifact (Polyman, version 1.153.1065) and eye blink and muscle artifact was removed by the B-Alert decontamination algorithms. Decontaminated data were then Fast Fourier Transformed into power spectra and power spectral densities were calculated for the following frequency bands; Alpha slow (αS) (8–10 Hz), alpha fast, (αF) (10–13 Hz), Beta (β) (13–30 Hz), and Gamma (30–40 Hz). Prior to statistical analysis EEG channels were divided into specific regions based on Brodmann's areas (Brodmann, 2006) aligning with 10–20 international EEG system as follows; Ventrolateral prefrontal cortex (VLPFC) F7 and F8, Dorsolateral prefrontal cortex (DLPFC) F3 and F4, Anterior prefrontal cortex (AntPFC) FP1 and FP2 and motor cortex (MC) C3, CZ, and C4. Both resting and exercise data were then calculated as percent changes from EC power spectral density baseline measures to correct for day-to-day and between-subject variability as previously reported (Nielsen et al., 2001; Bailey et al., 2008).

#### NIRS analysis

Changes in concentration of Oxyhemoglobin (*OHb*) and De-Oxyhemoglobin (*HHb*) were calculated using the modified Beer-Lambert law, based on changes in light attenuation at three wavelengths and the adult forehead differential path length factor of 5.93 (Van der Zee et al., 1992). Tissue Oxygenation Index (TOI) and normalized Tissue Hemoglobin Index (nTHI) were measured by the Spatially Resolved Spectroscopy (SRS) method. The TOI is a percentage ratio of *OHb* to Total Hemoglobin (*THb*) and represents the oxygen saturation of tissue hemoglobin. The nTHI is the sum of *OHb* and *HHb*, expressed as a ratio, which represents the relative concentration of total tissue hemoglobin and is, therefore, an indicative measure of regional blood volume (Van Beekvelt et al., 2001). Following testing, data were imported into an excel spreadsheet for normalization to the baseline measure. Each parameter was averaged for the period of the data collection (60 s during rest, 30 s during exercise). These averaged data were then normalized as percentage change from baseline.

#### Salivary cortisol analysis

Salivary cortisol concentrations were determined using commercially available high sensitivity enzyme immunoassay (ELISA) kits (Salimetrics, State College, PA, USA). Samples were defrosted, vortexed, and centrifuged prior to starting the analysis process. The intra-assay coefficient of variation for the duplicate samples was 7.8% across five plates and 190 samples run in duplicate.

### Statistical analysis

Due to injury, one participant had be excluded from the exercise sessions, therefore, the data are reported as *n* = 10, unless otherwise stated. All data were tested for normal distribution using a Shapiro-Wilks test. Data were analyzed using three and two way General Linear Model Repeated Measures ANOVAs.

Resting and exercise NIRS and EEG data were analyzed initially using a three way ANOVA for drug × hemisphere × time. Resting EEG were analyzed dependent on the channels in question. For the PFC, the channels were divided into regions (ANTPFC, DLPFC, VLPFC) and run as a three way ANOVA for drug × region × time. In the instance of exercise EEG all data were analyzed as a two way ANOVA for drug × time, due to removal of some subject's data, meaning that it was not possible to compare regions with each other without losing statistical power. Following a significant *F*-test, pair-wise differences were identified using a Bonferoni *post-hoc* test. One way ANOVAs with Tukey's correction for multiple comparisons *post-hoc* test was used to locate differences when a significant main effect or interaction effect was identified. Significance was set at *P* < 0.05. Results are presented as means ± *SD*.

## Results

### Cortisol awakening response

Salivary cortisol levels (nmol/l) significantly increased from awakening (0) to 30 min (13.3 ± 5.04 and 29.04 ± 9.5, respectively; *P* < 0.01). When represented as area under the curve relative to ground (AUC_G_), cortisol response was significantly increased at all time points with T30 being significantly higher than T45 and T60 (852.9 ± 211.3, 624.1 ± 192.7, and 504.5 ± 265.0, respectively; *P* < 0.05).

### Resting measurements

#### Salivary cortisol responses

**Figure 2:**
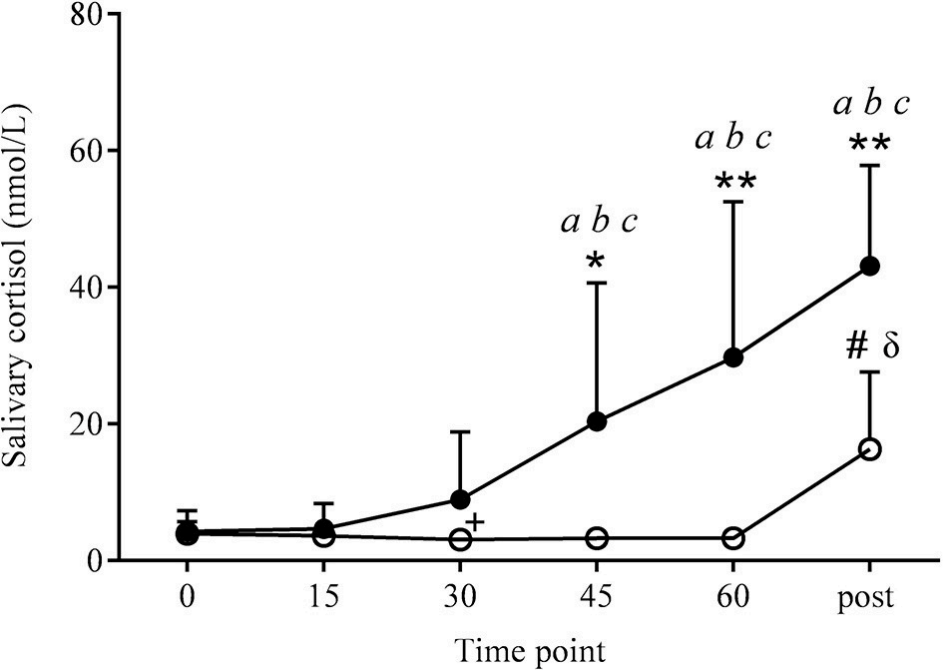
**Salivary cortisol responses at rest and 10 min post-exercise**. Values were significantly higher in COR (•) than PLA (o) at the same time point, at 45 (^*^P < 0.05), 60 and post-exercise (^**^P < 0.01). Salivary cortisol levels significantly increased across the COR trial, being higher at 45, 60, and post-exercise compared to 0 (^*a*^*P* < 0.05), 15 (^*b*^*P* < 0.05), and 30 (^*c*^*P* < 0.01). PLA increased significantly at post-exercise (^#^*P* < 0.01) compared to all other time points in the PLA condition. Post-exercise PLA values were also higher than COR at 0 and 15 min (^δ^*P* < 0.01). PLA decreased from 0 to 30 min (^+^*P* < 0.01).

Peak salivary cortisol values were significantly higher (*P* = 0.001) in COR than PLA (33.8 ± 21.3 and 4.2 ± 1.7 nmol/l, respectively; see Figure 2). There was also a significant effect of time (*P* < 0.001) and a drug × time interaction (*P* < 0.001). *Post-hoc* analysis revealed COR to be higher than PLA at 45 and 60 (20.4 ± 20.3 vs. 3.3 ± 1.2, 29.8 ± 22.8 vs. 3.3 ± 0.7 nmol/l, respectively; *P* < 0.05 and *P* < 0.01, respectively). Salivary cortisol in the COR trial significantly increased over time with both 45 and 60 being higher than 0 (45 and 60 min vs. 0; *P* < 0.05), 15 (45 and 60 min vs. 15; *P* < 0.05), and 30 (45 and 60 min vs. 30; *P* < 0.01). Values in the PLA condition actually decreased over time with 30 min being significantly lower than 0 (*P* < 0.01).

### Neurophysiological measures

#### Electroencephalography

##### Prefrontal cortex

There was a significant effect of the drug on the bandwidth response in the ANTPFC and the VLPFC (*P* < 0.05) for the COR to be higher than the PLA trial, but not the DLPFC.

#### Bandwidth response: alpha slow (αS)

##### Anterior prefrontal cortex

**Figure 3:**
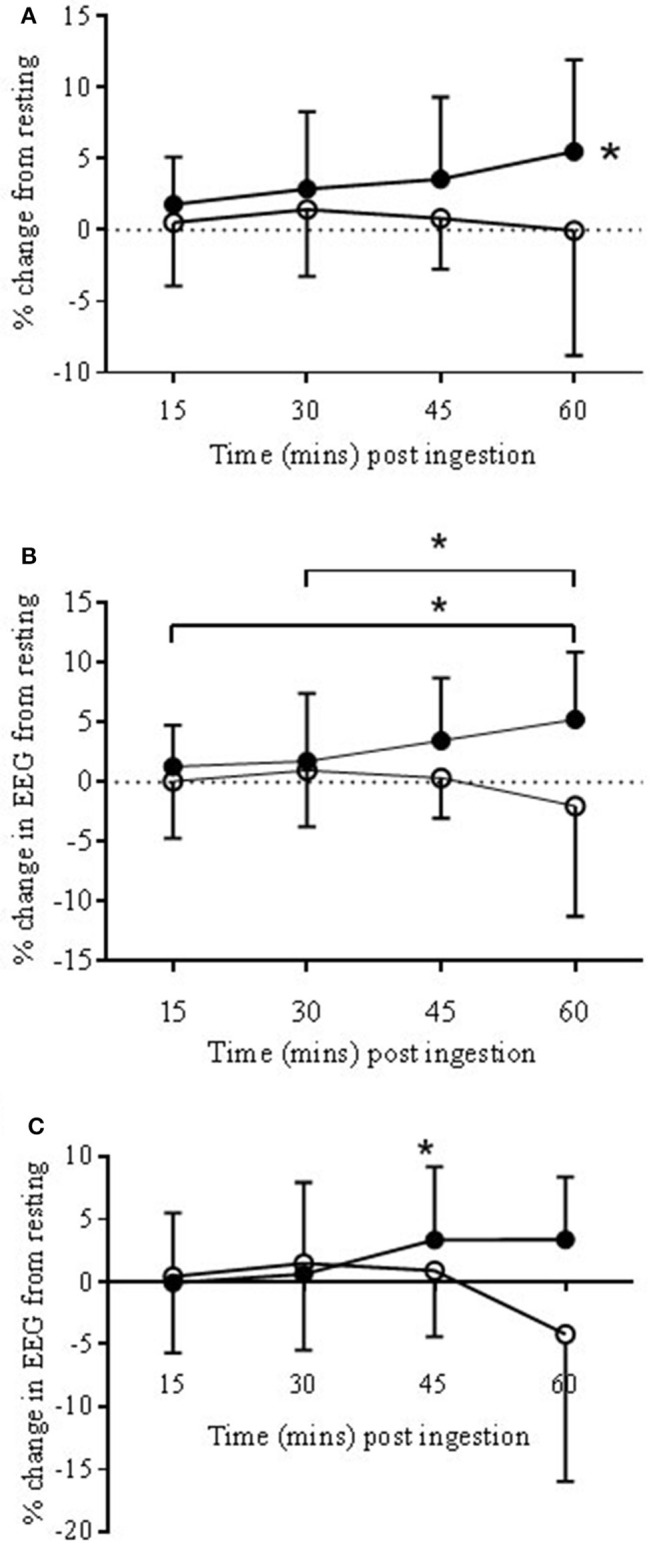
**EEG Alpha slow (αs) bandwidth responses to COR (•) and PLA (o) in ANTPFC (A)**, VLPFC **(B)**, and MC **(C)** during rest presented as % change from rest (0 min) to 15, 30, 45, and 60 min post-ingestion. **(A)**: The COR trial was significantly higher in the ANTPFC (^*^P < 0.05) compared to PLA. **(B)**: αS increased across time with 60 min being significantly higher than both 15 and 30 min (^*^P < 0.05). **(C)**: In the MC αS increased in the COR trial from 30 to 45 min (^*^P < 0.05).

Within the ANTPFC, αS was significantly higher, on average, in the COR trial compared to PLA (3.67 ± 5.28 vs. 1.0 ± 5.79%, respectively, *P* < 0.05; see Figure 3A).

##### Ventrolateral prefrontal cortex

Within the VLPFC αS was significantly higher, on average, in the COR trial compared to PLA (2.96 ± 5.13 vs. −0.13 ± 5.82%, respectively; *P* < 0.05; see Figure 3B). There was also a trend for a significant drug × time interaction (*P* = 0.067). *Post-hoc* analysis revealed significant increases from 15 and 30 to 60 min post-ingestion (*P* < 0.05).

#### Bandwidth response: gamma (γ)

##### Anterior prefrontal cortex

**Figure 4:**
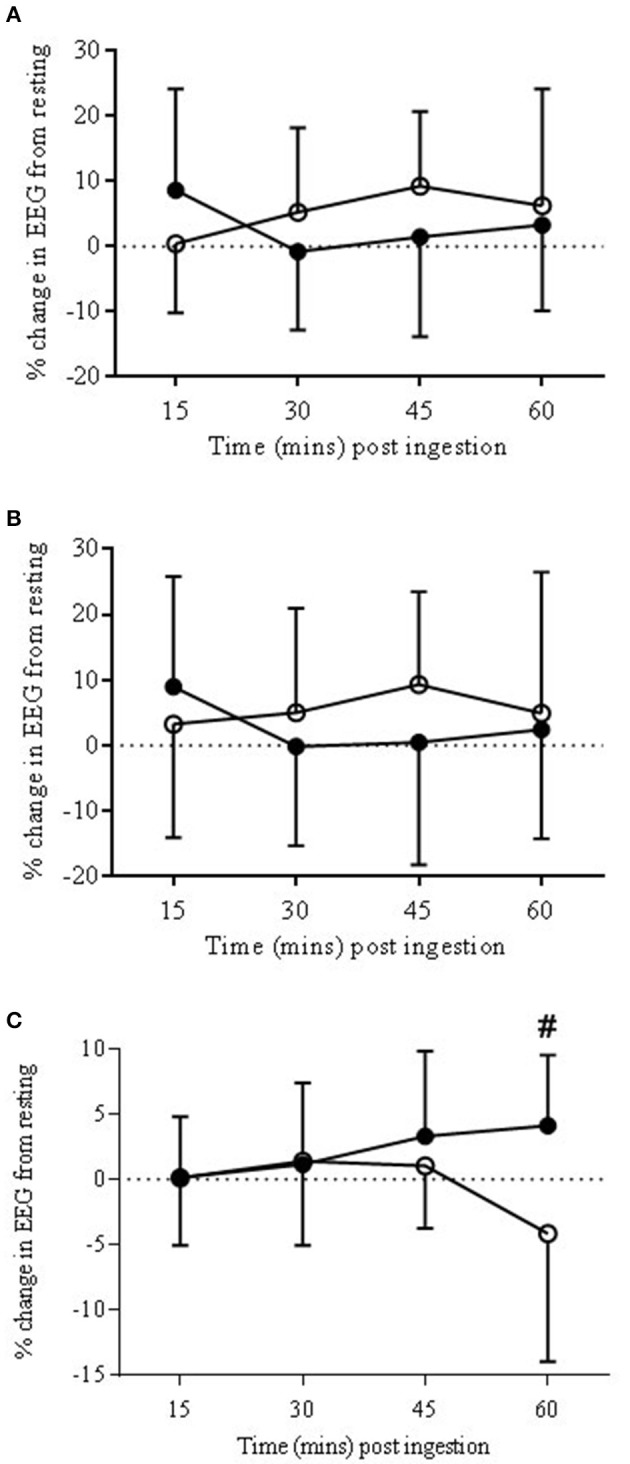
**EEG Gamma bandwidth responses to COR (•) and PLA (o) in ANTPFC (A)**, VLPFC **(B)**, and MC **(C)** during rest. Presented as % change from rest (0 min) to 15, 30, 45, and 60 min post-ingestion. There was a trend for percentage change at COR 60 (3.88 ± 5.1%) to be higher than COR 15 (0.14 ± 6.2%) (^#^*P* = 0.07).

There was a trend for a significant drug × time interaction (*P* = 0.06) with the bandwidth γ. *Post-hoc* analysis found no significant differences between the two drug conditions (Figure 4A).

##### Ventrolateral prefrontal cortex

There was a significant drug × time interaction (*P* < 0.05). *Post-hoc* analysis found a significant (*P* < 0.05) change across time with COR 60 min (2.4 ± 16.6%) being lower than 15 (8.9 ± 16.8%), and higher than 30 (−0.2 ± 15.5%) but with no significant changes to the PLA trial (Figure 4B).

#### Motor cortex

##### Bandwidth response: alpha slow (αS)

There was a trend for a significant drug × time interaction (*P* < 0.067). *Post-hoc* analysis showed a significant increase from 30 to 45 min (0.6 ± 6.1 vs. 3.3 ± 5.8%) in the COR trial (*P* < 0.05; see Figure 3C) but with no significant changes in the PLA trial.

##### Bandwidth response: gamma (γ)

There was a trend for a significant drug × time interaction (*P* = 0.06). There was also a trend for percentage change at COR 60 (3.88 ± 5.1%) to be higher than COR 15 (0.14 ± 6.2%; *P* = 0.07; see Figure 4C) but with no significant changes in the PLA trial.

There were no significant differences across the resting period in αF or β responses.

### Hemispherical differences

There were no hemispherical differences between COR and PLA suggesting that changes to neurophysiological measures were global or regional rather than the drug effecting one hemisphere of the brain differently than the other.

### Near infrared spectroscopy

There were no significant differences in NIRS responses in any of the four parameters measured; OHb, HHb, nTHI, and TOI. There was a trend (*P* = 0.07) for an effect of drug in HHb suggesting COR may result in HHb compared to PLA.

### Blood measures

There were no significant differences between baseline measures and 60 min post-ingestion in blood glucose responses in either COR or PLA (see Table 1). Blood glucose values were also assessed for change across time within individuals, but these were not significant. From resting (0 min) to 60 min post-ingestion, change in blood glucose in the COR and PLA trial was −0.2 ± 0.9 and −0.1 ± 0.4 nmol/l, respectively.

**Table 1:** **Plasma lactate and glucose responses during rest and exercise in mmol.L^−1^ as mean ± *SD* following ingestion of cortisol (COR) or placebo (PLA)**.

	**Blood lactate (mmol/L)**		**Blood glucose (mmol/L)**	
	**COR**	**PLA**	**COR**	**PLA**
Baseline	1.48±0.45	2.01±1.36	5.5±0.44	5.5±0.44
60 min post-ingestion	1.67±0.76	2.66±0.54	5.4±0.36	5.4±0.36
BL 1	9.3±2.6^*^	9.8±4.6^*^	5.9±1.36	6.46±0.8
BL 2	9.8±4.2^*^	9.9±4.6^*^	6.7±1.05^&^	7.1±1.2^&^

Significant effect of time with exercise lactate levels being significantly (

* P < 0.01) higher than both baseline and 60 min post-ingestion. Blood glucose responses were significantly higher at exercise 2 compared to 60 min post-ingestion (

& *P < 0.01)*.

### The effect of cortisol ingestion on BART test performance

One way ANOVAs with Dunnett's multiple comparisons test was used to test differences between the BASE and the COR and PLA in the following parameters of the BART test; Pumps adjusted average, Explosions (total), pumps adjusted average (pumps 21–30), pumps adjusted total and total pumps (not adjusted for explosions). One subject was removed due to improper completion of the test.

**Figure 5:**
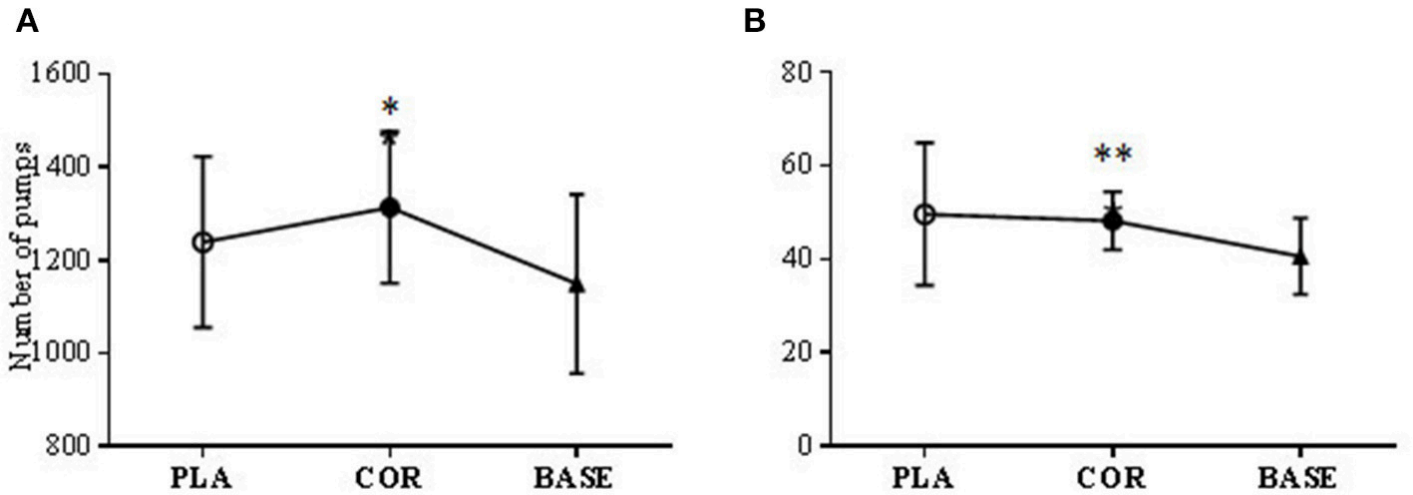
**BART test number of pumps (A)** and number of adjusted pumps **(B)** in cortisol (COR), placebo (PLA), and baseline (BASE). The COR trial was significantly higher than BASE in the total number of pumps made **(A)** (^**^P < 0.01) and the number of pumps made adjusted for the number of bursts **(B)** (^*^P < 0.05).

Two significant differences were found. Pumps adjusted average for COR was significantly higher (*P* < 0.05) compared to the BASE trial (1313.5 ± 162.9 vs. 1149 ± 192.5) but not PLA (1239 ± 183.7). Total pumps altogether (not adjusted for explosions) was also significantly higher in COR than the BASE trial (48.2 ± 6.2 vs. 40.6 ± 8.1; *P* < 0.01; see Figure 5).

No other differences were found between the BASE trial and either COR nor PLA.

### Exercise data

#### Exercise performance

**Figure 6:**
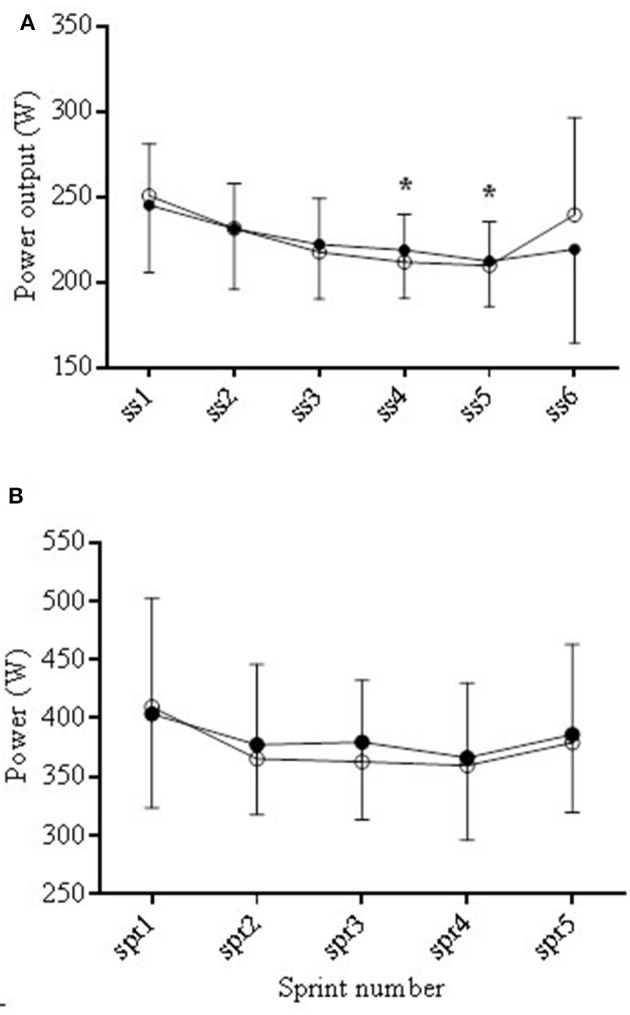
**Average power output (PO) during the COR (•) and PLA (o) trials during the steady state (SS) periods (A)** and the sprints **(B)**. During steady state PO decreased from SS2 to SS4 and SS5 (^*^P < 0.05).

All average performance data are shown in Table 2. There were no significant differences between distance covered, total average power output, average sprint and average steady state power output between trials. During steady state, PO decreased from SS2 to SS4 and SS5 (^*^P < 0.05) but with no difference between trials (see Figure 6). Peak power obtained during each trial was significantly lower (*P* < 0.05) in COR compared to PLA (461.4 ± 87.8 and 499.8 ± 104.3 W), respectively (Table 2).

**Table 2:** **Performance data from each 30 min trial**.

**Condition**	**Distance covered (km)**	**Average power output (W)**	**Average steady state power output (W)**	**Average sprint power output (W)**	**Peak sprint power output (W)**
COR	17.45 ± 0.65	237.1 ± 27.43	227.1 ± 16.4	382.6 ± 65.72	461.4 ± 87.8^*^
PLA	17.52 ± 0.76	237.1 ± 20.64	225 ± 11.7	375 ± 75.73	499.8 ± 104.3

* *Significantly lower (P < 0.05) than PLA*.

#### Rating of perceived exertion (RPE)

Mean RPE was not significantly different between COR (16.7 ± 1.98) and PLA (16.9 ± 1.89). There was a significant effect of time (*P* < 0.001), with RPE at each time point being higher than RPE 1. There was also an increase between RPE 2 and RPE 3 (*P* < 0.05) and RPE 2 and RPE 5 (*P* < 0.05).

#### Blood markers

There was a main effect of time (*P* < 0.01) with blood lactate being higher during exercise at both time points compared to both resting samples (see Table 1). Blood glucose responses were significantly higher at BL 2 compared to 60 min post-ingestion (*P* < 0.01; see Table 1). There were no differences between conditions in either measure.

#### Heart rate

Heart rate (HR) significantly increased during exercise in both steady state and sprint periods (*P* < 0.001). There was no difference between trials. HR in the steady state (SS) periods increased in the COR and PLA trials from 144.8 ± 17.1 to 175.1 ± 13.3 and from 141.9 ± 16.6 to 175.1 ± 14.7. During the (SS) periods each value was significantly (*P* < 0.05) higher than the previous one except between points 4 and 5. During the sprints average HR also increased significantly for consecutive time points.

### Neurophysiological measurements

#### Electroencephalography (EEG)

Although EEG responses were altered at rest by COR, these differences did not maintain significance during exercise. Due to artifact the full complement of channels and subjects could not be analyzed. The MC (*n* = 10) and VLPFC (*n* = 7) were analyzed as previously described. Channel FP1 (*n* = 8) was analyzed in representation of the ANTPFC and Fz (*n* = 7) was analyzed in place of the DLPFC.

Data were calculated as percentage change from baseline and analysis run with all five exercise time points and the final resting time point (60) to give a reference point of change from resting measures.

#### Prefrontal cortex

Within all regions of the PFC, there was a significant effect of time.

#### Anterior prefrontal cortex (FP1)

There was a significant effect of time in FP1 in αS, αF, β, and γ (*P* < 0.05, *P* < 0.01, *P* < 0.001, *P* < 0.001, respectively). *Post-hoc* analysis showed the differences across time to be from 60 min to all exercise time points (*P* < 0.05).

#### Ventrolateral prefrontal cortex

There was a significant effect of time in VLPFC in αS, αF, β, and γ (*P* < 0.05, *P* < 0.01, *P* < 0.001, *P* < 0.001, respectively). *Post-hoc* analysis showed the differences across time to be from 60 min to all exercise time points (*P* < 0.05) in all bandwidths except αS. In αS there was a trend for a difference between exercise time points 1 and 3 (*P* = 0.09).

#### Dorsolateral prefrontal cortex (Fz)

There was a significant effect of time in Fz in αS, αF, β, and γ (*P* < 0.001, *P* < 0.001, *P* < 0.001, *P* < 0.001, respectively). *Post-hoc* analysis showed the differences across time to be from 60 min to all exercise time points (*P* < 0.01) in all bandwidths.

#### Motor cortex

There was a significant effect of time in MC in αS, αF, β, and γ (*P* < 0.001, *P* < 0.001, *P* < 0.001, *P* < 0.001, respectively). *Post-hoc* analysis showed the differences across time to be from 60 min to all exercise time points (*P* < 0.001) in all bandwidths.

There was a significant decrease in β from exercise time point 2–5 (141.2 ± 11.8–131.2 ± 9.5; *P* < 0.05) showing a decrease during exercise toward the end.

### Near-infrared spectroscopy (NIRS)

#### Steady state

**Figure 7:**
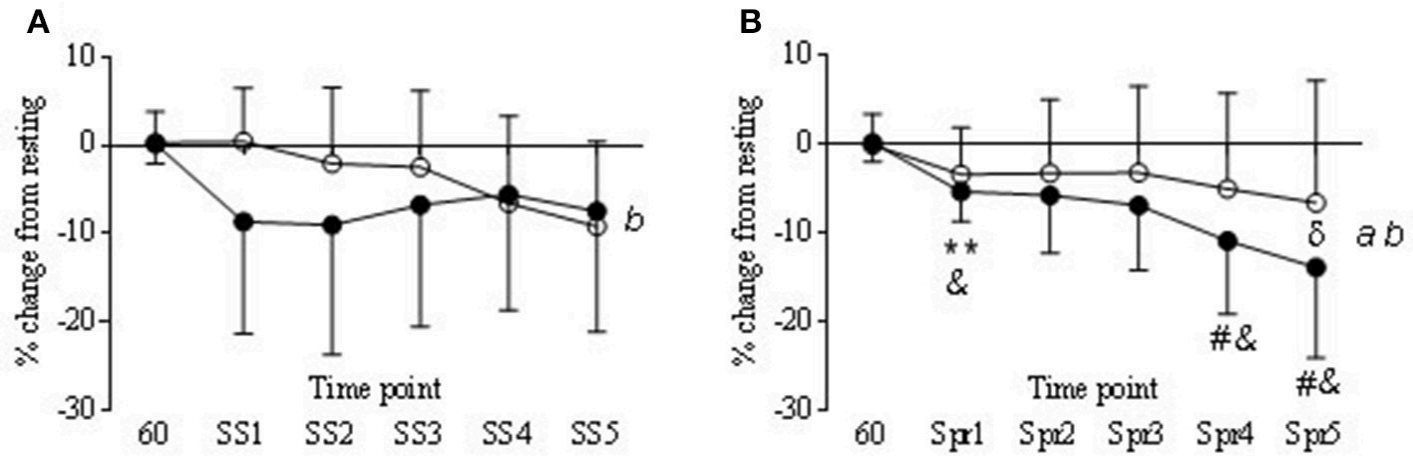
**Tissue oxygenation responses (TOI) in COR (•) and PLA (o) as % change from rest (60 min) to each steady state (SS) (A)** and Sprint (Spr) **(B)** interval. Main effect of drug (a) and time (b). Significant differences in COR from 60 to Spr 1 (^**^P < 0.01). Trend for COR Spr 4 and 5 (^#^*P* = 0.06 and 0.07 respectfully) to be lower than COR 60. COR significantly lower than PLA 60 at Spr 1, 4, and 5 (^&^*P* < 0.05). Significant difference between COR and PLA at Spr 5 (^δ^*P* < 0.05).

**Figure 8:**
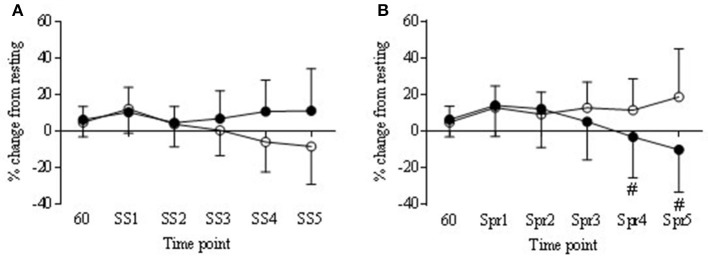
**Normalized tissue hemoglobin (nTHI) in COR (•) and PLA (o) as % change from rest (60 min) to each steady state (SS) (A)** and Sprint (Spr) **(B)** interval. Trend for COR Spr 4 and 5 (^#^*P* = 0.09 and 0.07 respectfully) to be lower than PLA.

During the steady state periods there was a significant decrease from baseline in TOI in both COR and PLA trials (*P* < 0.05) although in the COR trial TOI was lower to start with (Figure 7), this was not significantly different to PLA. There was a significant drug × time interaction in nTHI (*P* < 0.01) with COR higher on average than PLA (8.78 ± 2.8 and 0.41 ± 8.2, respectively) but *post-hoc* analysis revealed no significant differences between trials (Figure 8A). OHb was significantly increased during exercise in both COR and PLA (*P* < 0.001) reaching 3765 ± 2435 and 3458 ± 1634% change by SS 5, respectively. While HHb increased in COR (12787 ± 27418% change) and remained fairly level in PLA (1603 ± 1724% change), these differences were not significant.

#### Sprints

Tissue oxygenation index (TOI) decreased over time in the COR trial, being significantly lower at Spr 1 compared to 60 min (−5.3 ± 3.3 vs. 0.18 ±2.1) and a trend to be lower than 60 min at Spr 4 and 5 (−10.8 ± 8.2 and −13.9 ± 10.2, respectively; *P* < 0.05). Levels of TOI were lower in COR compared to PLA at Spr 5 (−13.9 ± 10.2 vs. −6.5 ± 13.7, respectively; *P* < 0.05; Figure 7B). There was also a decrease in the COR trial at Spr 1, 4, and 5 compared to PLA 60 (*P* < 0.05), whereas, there were no changes across the PLA trial that reached significance. nTHI also decreased across the COR trial, which whilst was not significant reached a trend for significance at Spr 4 and 5 (*P* = 0.09 and 0.07; see Figure 8B). As with the SS, OHb was also significantly increased from 60 to Spr 2–5 (*P* < 0.01) in both conditions with a trend for an increase from 60 to Spr1 (*P* = 0.07). There were no significant differences between conditions in HHb.

### Salivary cortisol responses post-exercise

Salivary cortisol values were significantly higher in COR than PLA post-exercise (43.1 ± 14.8 vs. 16.3 ± 11.3 nmol/l, respectively; *P* < 0.01). Post-exercise measures in COR were higher than 0 (*P* < 0.05), 15 (*P* < 0.05), and 30 (*P* < 0.01). PLA only increased over time at post-exercise with this time point being significantly higher than all other time points (*P* < 0.01). Salivary cortisol levels in the PLA trial post-exercise were also significantly higher than COR 0 and 15 (0 and 15 min; *P* < 0.01).

## Discussion

Ingestion of exogenous cortisol significantly altered resting measures of salivary cortisol and also EEG responses in both the alpha slow and gamma bandwidths. These changes were related to an increase in risk taking behavior as shown by the change in the number of pumps made in the BART test whilst under the influence of higher cortisol levels. During exercise, peak power production was lower in the COR trial. Further differences during the COR trial, compared to PLA, were identified in exercise responses of TOI and nTHI during sprinting which were lower than the PLA trial. There were no other differences in exercise performance.

### Changes in salivary cortisol levels

Salivary cortisol levels were successfully elevated with the COR condition over the 60 min rest following ingestion. Previous studies using exogenous cortisol have found similar levels as these (Buchanan and Lovallo, 2001; Henckens et al., 2012) although higher levels have also been reported with similar protocols (Putman et al., 2007, 2010). In the PLA trial salivary COR showed a decline from initial baseline levels during rest. This has been shown previously and likely reflects normal diurnal variation or possibly habituation to the slight initial stress of coming into the lab for a study (Putman et al., 2010). Despite the presence of elevated cortisol levels prior to exercise, further cortisol release was still stimulated from the adrenal cortex but there did not seem to be a relationship between elevated cortisol levels and an impact on exercise performance.

### Risk taking behavior

The BART test provides a sequential assessment of risk taking. There was one significant change in the number of pumps made in the COR trial compared to the BASE. This suggests that in the COR trial subjects made more pumps in an attempt to win more money. However, there were no changes between groups in terms of overall performance or in pacing strategy that was suggestive of an altered perception of risk. Previous research has shown that risk perception can impact on initial pacing strategy (Micklewright et al., 2015), however, we did not find that this was modulated here.

In previous research showing motivated decision making being increased by COR in some studies the participants had much higher COR levels following a 40 mg dose than was shown in the present study; 116.4 nmol/l 1 h after administration (Putman et al., 2010) vs. 29.7 ± 22.7 nmol/l at 60 min post-administration and 33.8 ± 21.3 nmol/L as peak values in the present study. This difference may explain why we were not able to find more changes in risk related behavior in our participants. Changes in risk related behavior are also time dependent (Henckens et al., 2012) and although we aimed to measure delayed effects of exogenous cortisol, it is possible that some rapid effects remained in some individuals and thus changes in reward based decision making might have been impaired in this instance (Koot et al., 2013). Further to this, HPA axis physiology is determined by previous stressful events associated with hypercortisolism (Herman and Cullinan, 1997; de Carvalho Tofoli et al., 2011) and, therefore, it has been hypothesized that endurance-trained individuals might develop adaptive mechanisms such as decreased sensitivity to cortisol to protect muscle and other glucocorticoid-sensitive tissues against this increased post-exercise cortisol secretion (Duclos et al., 2003).

### Neurophysiological measures

There were significant alterations to the neural responses at rest in the EEG. Changes in neuronal excitability are known to occur with glucocorticoid receptor mediated events (Joëls and de Kloet, 1992) such as would have occurred with exogenous cortisol. An increase in alpha slow at rest in the COR trial, predominantly within the PFC at the ANTPFC and VLPFC occurred, but also in the MC. Our understanding of changes to alpha are varied and, therefore, interpretation of these data is complex. For example Alpha oscillations seen at the surface EEG occur when there is disengagement of underlying cortical systems in active processing (Coan and Allen, 2004) also described as inhibition of neural ensembles (Uusberg et al., 2013). On the other hand it is generally held that alpha oscillations are seen in surface EEG if neurons are synchronously active across several centimeters of cortex (Pfurtscheller and Lopes da Silva, 1999; Nunez et al., 2001).

The presence of alpha has also been proposed to relate to metabolism in the area in which it is measured. The increase in alpha may, therefore, reflect a change in metabolism as a result of the presence of the COR. Previous research has attempted to examine the change in the blood oxygen level dependent (BOLD) signal in conjunction with changes in frequency of alpha bandwidth. Increased alpha activity has been shown to coincide with decreases in blood flow to the same area (Oakes et al., 2004) and to positively correlate with glucose metabolism and activity in the thalamus (Schreckenberger et al., 2004) suggesting a close functional relationship between thalamic activity and alpha rhythm. These studies have predominantly examined changes in the Occipital lobe created with eyes closed alpha and, therefore, may make it difficult to apply these theories to changes at the PFC which have been shown to relate to attention and motivational affective responses (Uusberg et al., 2013). However, desynchronization has also been found in both frontal and parietal lobes to have greater BOLD signal, that is greater metabolism with less alpha present (Laufs, 2003).

Changes in the EEG response at rest with exogenous COR may, therefore, reflect a change in the areas of the brain that are metabolically active which might be in conjunction with increased activity of thalamic areas. The negative feedback role of cortisol on the brain has been proposed to directly inhibit the core structures of the HPA axis (Herman and Cullinan, 1997) which would support a cause for changes in thalamic metabolism. If an increase in alpha is reflective of inhibition or decoupling of neural networks then this may explain the EEG changes seen here, where by the cortisol binds to GRs and suppresses subsequent HPA activation. It may also be a reflection of changes in activity within parts of the PFC involved in the HPA axis response and feedback loop which might be expected (Crane et al., 2003; Joëls et al., 2012).

During exercise the COR did have a significant impact on cerebral oxygenation responses, predominantly during the sprints where TOI signal declined over time throughout the sprints. This continual decline in oxygenation during repeated sprints is not uncommon (Racinais et al., 2007; Smith and Billaut, 2010; Smith et al., 2012) even though more mechanical work is not produced to prompt a greater demand in brain circulation or metabolism. The decline in nTHI would seem to indicate a reduced blood volume in that area and in relation to the changes of TOI, the percentage of OHb within the blood flowing to that region was less, suggesting a greater level of desaturation via higher oxygen uptake (González-Alonso et al., 2004). This is supported by the fact that HHb was higher in the COR trial, although not significantly so. Higher HHb may indicate a greater level of deoxygenation in the underlying tissue (Billaut et al., 2010) which further suggests that the presence of COR created a demand which lowered blood volume at the PFC and also desaturated the PFC to a greater extent. However, these events did not impact on exercise performance.

Oxygen availability did not change during the exercise period as OHb remained elevated in both trials throughout exercise. As power output was not affected by either condition this is in support of previous findings that suggest maintenance of oxygen availability does not impact on power output (Rooks et al., 2010; Smith and Billaut, 2010). Although other changes in NIRS measures may indicate a change in metabolism, oxygen availability remained high which is in agreement with previous findings that performance levels are maintained in this instance (Rooks et al., 2010). These changes in NIRS are in support of previous work showing that during self-paced exercise changes in cerebral oxygenation remain within a range that does not hinder exercise performance (Billaut et al., 2010) or conversely that changes in these parameters are not an important indicator for exercise regulation.

### Decreased peak power output

Despite decreases in peak power during exercise in the COR trial, overall TT performance was not affected between the two conditions. This, therefore, emphasizes the need to examine several aspects of performance when manipulating neurotransmitter responses. Previous manipulations of the dopaminergic pathways during exercise have only shown changes in time trial performance in the heat rather than in normothermic environments (Watson et al., 2005; Roelands et al., 2008). This may be due to the role of the hypothalamus in the manipulation of these pathways and its role in thermoregulation. In addition to this, previous research interrupting the HPA axis prior to exercise with ACTH dose has not shown any effects on maximal cycling performance (Soetens et al., 1995). However, having found a change in peak power output with elevated cortisol levels but no change in overall exercise performance it may be prudent in future research to include examination of several exercise models when manipulating neurotransmitter responses.

## Limitations

We would like to highlight the limitations to our study which may impact on the interpretation of the results. We were unable to provide monetary rewards to allow for payments to be made at the end of the BART test. This means that essentially the participants did not stand to lose anything, which may have impacted on how they performed the test. The results may also be limited in their interpretation due the relatively small *n* of 9.

## Conclusion

In conclusion, ingestion of exogenous cortisol was related to clear EEG changes at rest and NIRS changes during exercise. These changes were in conjunction with altered risk taking behavior as measured by the BART test, but no change in overall exercise performance occurred. Therefore, exogenous cortisol altered cognitive behavioral responses to a risk task as has been previously shown (Putman et al., 2010). Although peak power was altered this was not sufficient to impact on overall TT performance.

## Author contributions

CR devised study design, carried out experimental measures, undertook data analysis, interpretation, and writing of manuscript. FM and MI assisted with study design, data interpretation, and drafting and finalizing of manuscript.

## Funding

Funding for this study was provided by a Charles Sturt University Higher Degree by Research Scholarship.

### Conflict of interest statement

The authors declare that the research was conducted in the absence of any commercial or financial relationships that could be construed as a potential conflict of interest.
